# Diagnostic value of saline infusion sonohysterography for detecting endometrial focal lesion

**DOI:** 10.11604/pamj.2019.33.211.16563

**Published:** 2019-07-16

**Authors:** Sanam Moradan, Sati Nik Darzi, Raheb Ghorbani

**Affiliations:** 1 Abnormal Uterine Bleeding Research Center, Semnan University of Medical Sciences, Semnan, Iran; 2 Semnan University of Medical Sciences, Semnan, Iran; 3 Social Determinants of Health Research Center, Semnan University of Medical Sciences, Semnan, Iran

**Keywords:** Abnormal uterine bleeding, saline infusion sonohysterography, uterine focal lesion, sonohystrography, transvaginal ultrasound, hysteroscopy

## Abstract

**Introduction:**

Different diagnostic tools are available to evaluate endometrial focal lesion such as hysteroscopy, sonohystrography and transvaginal ultrasound. The present study aimed to determine the diagnostic value of saline infusion sonohystrography (SIS) in diagnosis of intrauterine lesions in women with Abnormal Uterine Bleeding (AUB).

**Methods:**

This cross-sectional study recruited 100 married women with chief complain of AUB referred to gynecologic clinics at the Amir Al-Momenin hospital, Semnan, Iran from March 2014 to February 2016. All participants were in the reproductive age and post-menopausal period that showed abnormal endometrial thickness or endometrial focal lesions through transvaginal ultrasound. Participants underwent SIS, hysteroscopy plus focal lesion resection and endometrial biopsy in order. The gold standard was the histopathology of endometrial specimen reported by pathologist.

**Results:**

Mean±SD age of women was 41.2±11.3 years. To diagnose the overall focal lesions, sensitivity, specificity, positive predictive value (PPV) and negative predictive value (NPV) of the SIS were 79.6, 89.1, 89.6, and 78.8% respectively. These figures were 75.0, 87.5, 82.5 and 81.7%, respectively to diagnose polyps. The SIS sensitivity, specificity, PPV and NPV values to diagnose the myomas were 60.0, 97.8, 75.0, and 95.7% respectively.

**Conclusion:**

Findings show that, SIS probably is a proper method for detecting endometrial focal lesion including polyps and myomas. Future studies may help to define further advantages of this procedure.

## Introduction

Abnormal uterine bleeding (AUB) is one of the most common disorders in women at the childbearing age. The AUB can cause complications in personal and social life [1]. The AUB term refers to any change and irregularity of menstrual cycle and covers all changes in the duration, number, and amount of bleeding [2,3]. Although it has been estimated that more than 30% of referrals to the women health centers are related to the AUB; it is worth to notice that some patients may not be referred [1]. This fact might explain why the prevalence of the AUB in different studies had a range from 10 to 52% [4-6]. The AUB has numerous disadvantages on patients' health-related quality of life (HRQL) such as loosing concentration on the jobs [3], treatment costs [7] and iron storage deficiency [8]. The HRQL, as defined by the World Health Organization (complete personal, social, psychological and spiritual health), is frequently used regarding bleeding disorders, particularly AUB [9-12]. In the UK, the AUB has been considered as a cause of physical and psychosocial health problems and total quality of life as a result of the medical and paramedical effects [13] that highlights the need to assess the AUB early and with tools that are reliable and valid. There are different diagnostic tools to evaluate the endometrial focal lesions. The main and the most common tools are transvaginal sonography, sonohystrography and hysteroscopy. This variety of tools makes the first choice difficult and controversial. Transvaginal Sonography (TVS) is the first measure to diagnose abnormal endometrium proliferation in the AUB condition in the pre-and post-menopausal ages [14,15], while, Saline Infusion Sonohystrography (SIS) seems to be superior to TVS, for uterine pathologies [16]. The SIS is usually carried out by entering the sterile saline into uterine cavity [14] and should be done on specific days (days 3 to 7 of the menstrual cycle and the best is day 6^th^ that is close to the end of bleeding period), when the Endometrial Cavity is thin [17,18]. Diagnostic hysteroscopy with endometrial biopsy is gold standard [19,20], allows inside vision of the uterus to diagnose and treat the causes of abnormal bleeding [20]. The study by Kelelci *et al.* in Turkey, reported the sensitivity and specificity of the SIS (81.3 and 100%, respectively), to be higher than TVS (56.3 and 72%, respectively) and it is almost the same as hysteroscopy (87 and 100%, respectively) [21]. The high sensitivity (91.9%) and specificity (98.8%), positive predictive value (PPV) (97.1%) and negative predictive value (NPV) (96.5%) of the SIS for the AUB, in the study by Makris *et al.* endorsed the reliability of this method [15]. This study aimed to investigate the sensitivity, specificity, positive and negative predictive values of the SIS.

## Methods

**Participants**: in this cross-sectional study, 100 patients referred to Amir Al-momenin Hospital in Semnan, from March 2014 to February 2016, were selected according to the inclusion criteria. All of the participants were married, age ≥18 years, with a history of the AUB, stable vital signs plus suspected to the focal lesion in their uterine cavity (especially polyps, submucosal myomas, irregular endometrium, and adhesion), or endometrial thickness ≥12 mm in the non-menopause stage and at or above 5 mm in the post-menopause stage. The AUB after menopause was considered as any bleeding without hormonal therapy. The participants did not have any history of diabetes, hypertension, thyroid disease, pelvic inflammatory disease and uterine surgeries. They did not have any evidence of pregnancy.

**Measurements**: the first author as an experienced gynecologist did the TVS, the SIS, the hysteroscopy, focal lesion resection and endometrial biopsy for all participants. The TVS and SIS were carried out between 3^rd^and 7^th^ days of the menstrual cycle and in menopause women at the earliest possible date. A transvaginal transducer (15M.Hertz probe) was used to measure endometrial thickness, size and position of myomas and polyps. To implement the SIS, a sterile speculum was passed and the cervix was disinfected with Betadine solution. A flexible Foley catheter (children feeding tube catheters, length: 15 cm, diameter: 2 mm) with inflatable balloon (produced by Supa, Tehran, Iran) was inserted via the cervical canal until the uterine was found. At the proper position of the catheter, 10 ml of sterile saline solution (0.9%) were injected by 50 ml volume syringe into the uterine cavity slowly and the injection was continued to obtain optimal view of the endometrial cavity. This procedure was performed without using any local anesthesia or prophylactic antibiotic. All participants in the proliferative phase of menstrual cycle underwent diagnostic operative hysteroscopy, which contains a telescope (2.7-4 mm in diameter) inserted into the endometrium cavity under a general anesthesia. The hysteroscopy was performed under cervical dilatation, prophylactic antibiotic and two misoprostol vaginal tablets (six hours before operation). Focal lesion resection and endometrium biopsy (as the gold standard tests) were performed during the hysteroscopy.

**Ethical considerations**: the study was approved by the human ethics committee of Semnan University of Medical Sciences. All participants were informed about the study before signing a consent form, and we were reassured to the patient that his information would be confidential.

**Statistical analysis**: we analyzed data by SPSS 23.0. The SIS sensitivity, specificity, positive and negative predictive value and accuracy (the ability of a specific tool to represent the proportion of true positive and true negative results in the selected population) were calculated.

## Results

Mean±Standard Deviation (SD) age of women was 41.2±11.3 years. Table 1 shows other characteristics information of women. The mean±SD endometrial thickness in SIS was 9.2±3.2 mm. Polyp was the most common focal lesion found by the SIS (40.0%) and the hysteroscopy (47.0%). However, Myoma was detected only in eight percent of the participants using the SIS and in nine percent of the participants through the hysteroscopy. Table 2 shows the agreement between the findings of the three different methods and the pathological findings. Table 3 demonstrates the figures of the sensitivity, specificity, positive predictive value (PPV) and negative predictive value (NPV) of the three different diagnostic approaches for the uterine focal lesions (polyps, myomas), polyps or myomas. SIS showed a sensitivity of 79.6%, 75.0% and 60.0% for focal lesions, polyps and submucous myomas respectively whereas the specificity was reported 89.1% for focal lesions, 87.5% for polyps and 97.8% for submucous myomas.

**Table 1:** Demographic variables in the study group (n=100)

Age	Percentage (%)
Premenopause (18-38)	52
Perimenopause (39-51)	35
Postmenopause (≥52)	13
**Delivery method**	
Natural Vaginal Delivery	36
Caesarean section	27
NVD+ caesarean section	24
Infertile	13
**Duration of menopause (year)**	
1-5	76.9
6-10	15.4
>10	7.7
**Duration of the AUB (month)**	
<6	44
6-12	30
13-18	18
19-24	4
>24	4
**Patterns of AUB**	
Polymenorrhea	3
Menorrhagia	5
Metrorrhagia	20
Postmenopausal bleeding	13
Menometrorrhagia	36
Others	23
**Other variables**	**Mean±SD**
Gravidity	2.96±2.01
Parity	2.67±1.71
Living children	2.63±1.60
Abortion	1.08±0.85

NVD: Natural Vaginal Delivery

**Table 2:** Number of uterine abnormalities diagnosed using SIS, TVS and hysteroscopy in compared with the final diagnosis at pathology

Pathological finding			TVS		SIS		Hysteroscopy	
Abnormality		n	yes	no	yes	no	yes	no
**Polyps or myomas**	Yes	54	17	37	43	11	54	-
	No	46	21	25	5	41	2	44
**Polyps**	Yes	44	9	35	33	11	44	-
	No	56	19	37	7	49	3	53
**Submucous myomas**	Yes	10	2	8	6	4	9	1
	No	90	8	82	2	88	0	90

TVS: Transvaginal Sonography SIS: Saline Infusion Sonohystrography

**Table 3:** Comparative diagnostic value of SIS, TVS and hysteroscopy in the diagnosis of focal lesions (polyps or myomas), polyps and myomas in compared with the final diagnosis at pathology

Variable	Sensitivity (%)	Specificity (%)	Positive predictive value (%)	Negative predictive value (%)
**SIS**				
Polyps or myomas	79.6	89.1	89.6	78.8
Polyps	75.0	87.5	82.5	81.7
Myomas	60.0	97.8	75.0	95.6
**TVS**				
Polyps or myomas	31.5	54.3	44.7	40.3
Polyps	20.5	66.1	32.1	51.4
Myomas	20.0	91.1	20.0	91.1
**Hysteroscopy**				
Polyps or myomas	100	95.7	96.4	100
Polyps	100	94.6	93.6	100
Myomas	90.0	100	100	98.9

SIS= Saline Infusion Sonohystrography; TVS= Transvaginal Sonography

## Discussion

The present study confirmed that the SIS has good sensitivity and very good specificity, PPV and NPV to detect the endometrial focal lesion. However, its sensitivity, specificity, PPV and NPV were less than those of the hysteroscopy and better than the TVS. Reviewing the literature concerned about the capability of these methods to detect endometrial focal lesions was controversial. There are studies that reported sometimes higher and sometimes lower scores for these features in the SIS. The finding of the present study is more promising than the findings reported by Yildizhan *et al.* for the specificity of the SIS. Yildizhan *et al.* indicated satisfactory specificity for the SIS to find the endometrial lesion [22]. Aslam *et al.* with similar aims reached to 92.9% and 89.7% for the sensitivity and specificity respectively for the SIS [23]. Again, their findings were close to the findings of the present study. They proved excellent and very good scores for the SIS to detect endometrial focal lesions that is better than the score of the present study. In 2000, Dijkhuizen *et al.* provided excellent sensitivity (100%) and very good specificity (85%) for the SIS to detect the uterine lesions. The researchers concluded that the diagnostic accuracy of the SIS was higher than those of the TVS and the combination of the TVS and reserving saline infusion sonohystrography in patients with increased thickness (above 5 mm), or patients that endometrium could not be seen adequately by the TVS [24]. Even the findings reported by the Alborzi *et al.* were not supported by the findings of the present study. Alborzi *et al.* indicated the SIS had excellent records for the sensitivity (94.1%), specificity (95%), NPV (90%) and PPV (96%) [25]. These excellent records for the SIS to detect focal lesion in the uterus was repeated in the other [26,27].

On the other hand, there are studies that presented results in line with the results of the present study such as Ogutcuoglu *et al.* Ogutcuoglu *et al.* examined the sensitivity, specificity, PPV and NPV of the SIS in 100 clients to detect endometrial focal lesion. Their findings were approximately similar to the findings of the present study [28]. Gunes *et al.* evaluated 83 patients with the AUB and the results showed the SIS was a very accurate method to detect polyps. They reported very good figures for the sensitivity, specificity, PPV and NPV of the SIS [29], which is approximately in line with the results of the present study. While the present study indicated that the SIS has less sensitivity and specificity than those of the hysteroscopy, the results of Soares *et al.* study (2000) found out the same diagnostic accuracy as the gold standard for the hysteroscopy and the SIS to detect the polyps, and endometrial hyperplasia [30]. The Soares results were repeated in the findings reported by Epstein *et al.* and Chawla *et al.* study (2014) that showed the SIS and hysteroscopy have very good sensitivity to detect endometrial polyps and endometrial abnormalities [31,32]. De kroon *et al.* in their study have also indicated that the SIS is a valid and reliable diagnostic alternative to hysteroscopy and diagnostic hysteroscopy can be limited to inconclusive or failed SIS [33]. The greatest challenge of the SIS in the present study was the inability to perform direct biopsy and special tools, like NiGo device has been produced which is easy to perform and it provides sufficient tissue sample. Another limitation of this study was the use of children feeding tube catheters, rather than SIS catheters (Elliptoshpere).

## Conclusion

Findings show that, SIS probably is a proper method for detecting endometrial focal lesion including polyps and myomas. Future studies may help to define further advantages of this procedure.

### What is known about this topic

- Various methods are used for diagnosis of endometrial focal lesions;
- SIS is one of the diagnostic tools of endometrial focal lesion;
- In some study saline infusion sonohystrography has high efficiency in diagnosis of endometrial focal lesions that is very near to hysteroscopy.

### What this study adds

- Saline infusion sonohystrography probably is a proper method for detecting endometrial focal lesions including polyps and myomas;
- Saline infusion sonohystrography is a suitable method for diagnosis of endometrial focal lesions prior hysteroscopy, but it could not be as an alternative of hysteroscopy (sensitivity and specifity respectively 79.6%, 89.1% for overall endometrial lesions).

## Competing interests

The authors declare no competing interests.
